# A novel circular RNA, circMAML3, promotes tumor progression of prostate cancer by regulating miR-665/MAPK8IP2 axis

**DOI:** 10.1038/s41420-023-01750-1

**Published:** 2023-12-14

**Authors:** Zeng Zhenhao, Chen Ru, Cheng Xiaofeng, Yang Heng, Wang Gongxian

**Affiliations:** 1 Department of Urology, Jiangxi Provincial People’s Hospital, The First Affiliated Hospital of Nanchang Medical College, 330000 Nanchang, China; 2 Department of Urology, The First Affiliated Hospital of Nanchang University, 330000 Nanchang, China; 3 Department of Urology, Fujian Medical University Union Hospital, 29 Xinquan Road, 29, Gulou District, 350001 Fuzhou, Fujian P. R. China

**Keywords:** Prostate cancer, Non-coding RNAs

## Abstract

Many studies have now demonstrated that circRNAs are aberrantly expressed in cancer and are involved in the regulation of malignant tumor progression. However, the role of circMAML3 (hsa_circ_0125392) in prostate cancer has not been reported. circMAML3 was selected from public data through screening. The circMAML3 circular characterization was performed using Sanger sequencing, agarose gel electrophoresis assay, RNase R assay and actinomycin D assay. The expression of circMAML3 in prostate cancer tissues and cells was detected by qRT-PCR. In vivo and in vitro experiments were conducted to investigate the biological functions of circMAML3 in prostate cancer. Finally, the underlying mechanism of circMAML3 was revealed by qRT-PCR, luciferase reporter assay, miRNA Pulldown, RNA immunoprecipitation, western blotting, and rescue assay. Compared to normal prostate tissue and prostate epithelial cells, circMAML3 is highly expressed in prostate cancer tissues and cell lines. CircMAML3 overexpression promotes prostate cancer proliferation and metastasis, while knockdown of circMAML3 exerts the opposite effect. Mechanistically, circMAML3 promotes prostate cancer progression by upregulating MAPK8IP2 expression through sponge miR-665. Our research indicates that circMAML3 promotes prostate cancer progression through the circMAML3/miR-665/MAPK8IP2 axis. circMAML3 and MAPK8IP2 are upregulated in prostate cancer expression and play an oncogenic role, whereas miR-665 is downregulated in prostate cancer and plays an oncogenic role. Therefore, CircMAML3 may be a potential biomarker for prostate cancer diagnosis, treatment and prognosis.

## Introduction

The most recent statistical analysis by the American Cancer Society shows that the incidence of prostate cancer (PCa) is on the rise [1]. In 2023, it is estimated that there will be 288,300 new cases of PCa, which is the most commonly diagnosed cancer among men. Furthermore, it is expected to cause approximately 34,700 new deaths, as the second leading cause of cancer-related deaths among males [1]. The main treatment modalities for PCa in clinical practice include radical surgery, endocrine therapy, radiotherapy, and chemotherapy, all of which can provide effective treatment for patients with PCa. However, patients with PCa have a worse prognosis if they develop metastasis or progress to castration-resistant. Although patients with localized PCa have a high 5-year survival rate (close to 100%), the 5-year survival rate for metastatic PCa is only 31% [2]. The prognosis of patients with metastatic castration resistance is even worse, with a median survival time of only 13 months and a 5-year survival rate of approximately 15% [3]. Although advances in diagnostic and therapeutic technologies have improved patient prognosis, continuous exploration is still necessary [4]. Thus, further research is necessary to explore novel biomarkers and therapeutic targets to improve the diagnosis and treatment of PCa.

With the rapid development of high-throughput sequencing technology and bioinformatics algorithms, a type of RNA molecule has been discovered as a potential tumor biomarker, which is called circular RNA (circRNA). CircRNA is a type of single-stranded covalently closed RNA molecule, which is generated from precursor mRNA (pre-mRNA) through back-splicing [5]. Studies have shown that circRNA have multiple biological functions, including transcriptional regulation, competition with pre-mRNA splicing, interaction with proteins, acting as miRNA sponges, interacting with mRNA to affect its expression, and competing with mRNA binding proteins to influence mRNA translation; in addition to their non-coding roles in regulating gene expression, some circRNA were found to have the ability to translate proteins [6, 7]. A significant amount of aberrantly expressed circRNA have been identified in cancer, and they play indispensable roles in the development and progression of tumors as either oncogenes or tumor suppressors [8]. Just as c-FLIP plays a role in cancer [9], circRNAs have a significant impact on cancer. Recently, studies have identified several circRNA with abnormal expression in PCa, and it has been demonstrated that some circRNA can promote or inhibit the proliferation, migration, and invasion of PCa cells. For example, circSCAF8 promotes prostate cancer by sponges miR-140-3p and miR-335 to regulate LIF expression [10]. hsa_circ_0003258 binding to IGF2BP3 enhances the stability of HDAC4 to promote PCa progression [11]. hsa_circ_0063329 inhibits PCa proliferation and metastasis by binding miR-605-5p to regulate TGIF2 expression [12]. circEXOC6B inhibits PCa metastasis via enhance RBMS1 and HuR binding to further increase AKAP12 expression [13]. However, there are still many abnormally expressed circRNA waiting to be explored in prostate cancer.

In this study, based on VO et al. [14] differential abundance analysis of circRNAs in 25 matched normal and prostate cancer samples, we identified a previously unreported circRNA, circMAML3 (circBase ID: hsa_circ_0125392), which was found to be highly expressed in prostate cancer tissues and cell lines. We identified the role of circMAML3 in PCa through gain- and loss-of-function experiments. Mechanistically, circMAML3 is mainly localized in the cytoplasm and regulates Mitogen-activated protein kinase 8 interacting protein 2 (MAPK8IP2) expression through sponge miR-665 to promote PCa proliferation and metastasis.

## Results

### Discovery and identification of circMAML3 in Pca

**Fig. 1:**
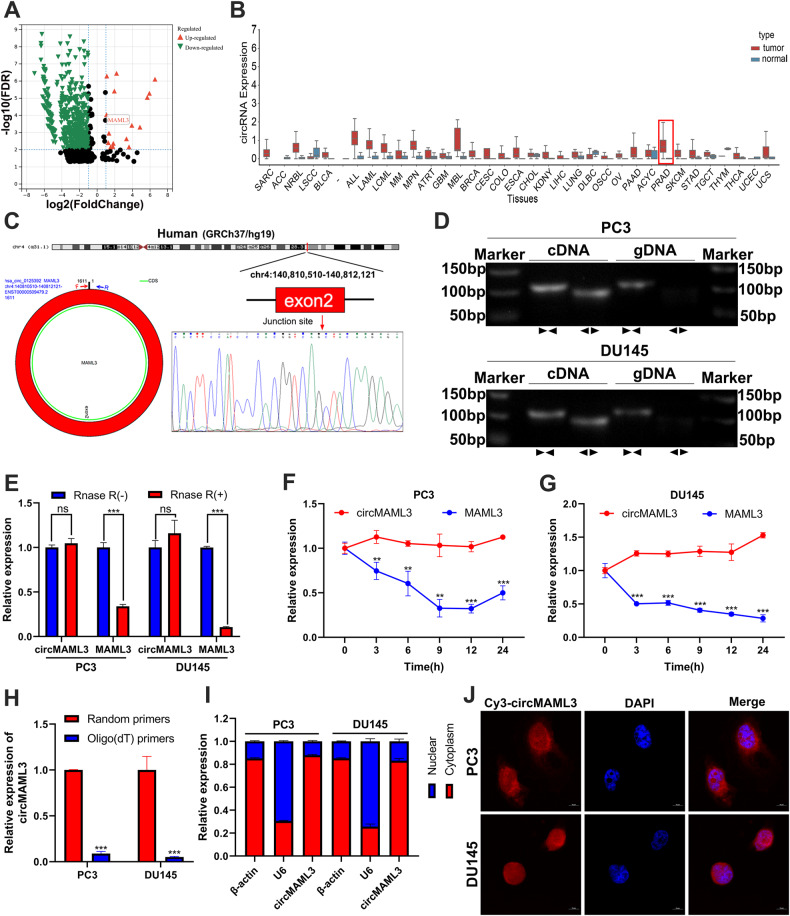
Screening, characterization and localization of circMAML3. **A** Differential analysis of circRNA volcano plots in 25 pairs of paired prostate cancer tissues versus normal tissues (logFC > 1, FDR < 0.01), with upregulated circRNAs in prostate cancer in red and downregulated cricRNAs in prostate cancer in green. **B** CircMAML3 expression in pan-cancer (including prostate cancer). **C** Schematic of circMAML3 circular structure and sanger sequencing analysis. **D** Agar gel electrophoresis assays to analyze the amplification of divergent primers (circMAML3) and convergent primers (MAML3 mRNA) in cDNA and gDNA. **E** qRT-PCR to detect the expression of circMAML3 and MAML3 in RNase R-treated versus untreated groups by RNA extracted from PC3 and DU145 cells. **F**, **G** Expression of circMAML3 and MAML3 mRNA was detected by qRT-PCR after treatment of PC3 and DU145 cells with actinomycin D at different time points. **H** cDNA templates obtained by reverse transcription by Random method and Oligo dT method, in which the expression of circMAML3 was detected by qRT-PCR. **I** qRT-PCR detection of circMAML3 for subcellular localization in PC3 and DU145 cells. **J** FISH assay to analyze circMAML3 localization in PC3 and DU145 cells. ** *P* < 0.01, *** *P* < 0.001.

According to the differential expression analysis of circRNA between PCa and normal tissues reported by VO et al. [14], we identified significantly upregulated circRNAs in prostate cancer tissues using the criteria of logFC > 1 and FDR < 0.01, and among them, circMAML3 was found to be present (Fig. 1A). In addition, the analysis using the circAtlas 2.0 (https://ngdc.cncb.ac.cn/circatlas/) [15] database revealed that circMAML3 was highly expressed in most cancer tissues compared to normal tissues, as well as in prostate cancer (Fig. 1B). circMAML3 (chr4:140810510-140812121) is formed by the reverse splicing of exon 2 of the parental gene MAML3, with a length of 1611 bp (Fig. 1C). To confirm the circular closed-loop structure of circMAML3, we employed specific divergent primers for amplification and subsequently validated it through Sanger sequencing in DU145 cells (Fig. 1C). Furthermore, we performed agarose gel electrophoresis on the qRT-PCR products generated using both divergent and convergent primers. The gel analysis revealed that circMAML3 exhibited amplification only in the cDNA samples derived from PC3 and DU145 cells, while no amplification was detected in the gDNA samples (Fig. 1D). We further confirmed that circMAML3 is more stable than linear MAML3 by treatment of PC3 and DU145 cells with RNase R and actinomycin D (Fig. 1E–G). Finally, in the qRT-PCR experiments of cDNA obtained by Random primer method and Oligo dT primer method, it was found that circMAML3 could be effectively amplified in the cDNA reversed by the Random primer method but not in Oligo dT primer method (Fig. 1H). To explore the subcellular localization of circMAML3 in PC3 and DU145 cells, we performed FISH and nuclear‑cytoplasmic fractionation assays, which showed that circMAML3 was mainly localized in the cytoplasm (Fig. 1I, J).

### CircMAML3 promotes PCa cell progression in vitro

**Fig. 2:**
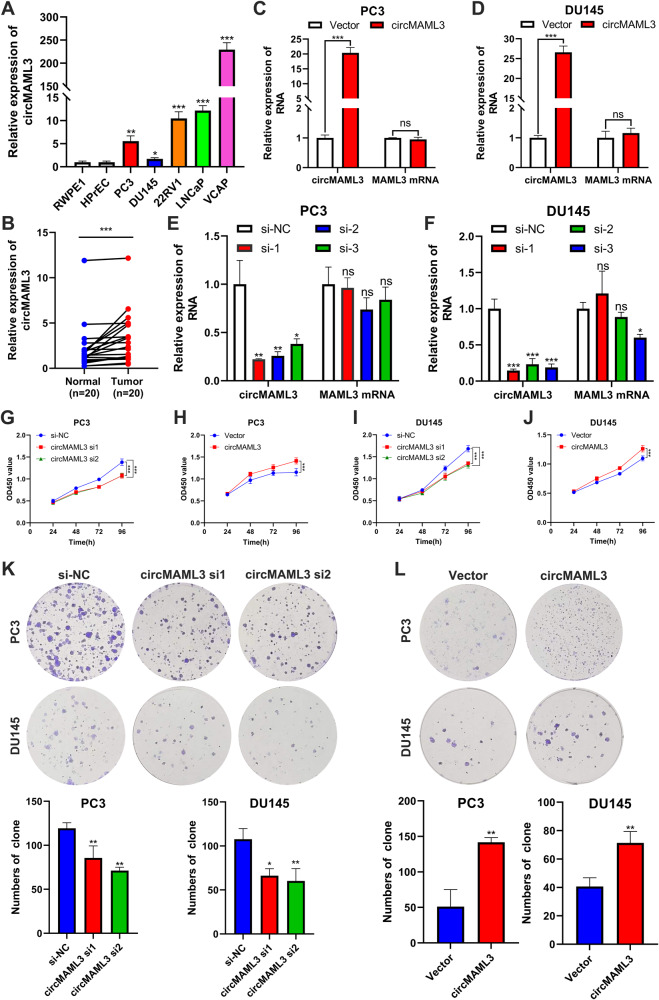
CircMAML3 expression in prostate cancer cells and tissues and its effect on prostate cancer cell proliferation, migration and invasion. **A**, **B** qRT-PCR assay revealed that circMAML3 is highly expressed in prostate cancer cell lines and prostate cancer tissues. **C**–**F** qRT-PCR to validate the overexpression (**C**, **D**) and knockdown (**E**, **F**) efficiency of circMAML3 in PC3 cells and DU145 cells. **G**–**I** CCK-8 assay to analyze the effect of knockdown and overexpression of circMAML3 on proliferation in PC3 cells (**G**, **H**) and DU145 cells (**I**, **J**). **K** Clone formation assay to assess the effect of the knockdown of circMAML3 on the proliferation of PC3 and DU145 cells. **L** Clone formation assay to assess the effect of overexpression of circMAML3 on the proliferation of PC3 and DU145 cells. ns: not significant, * *P* < 0.05, ** *P* < 0.01, *** *P* < 0.001.

**Fig. 3:**
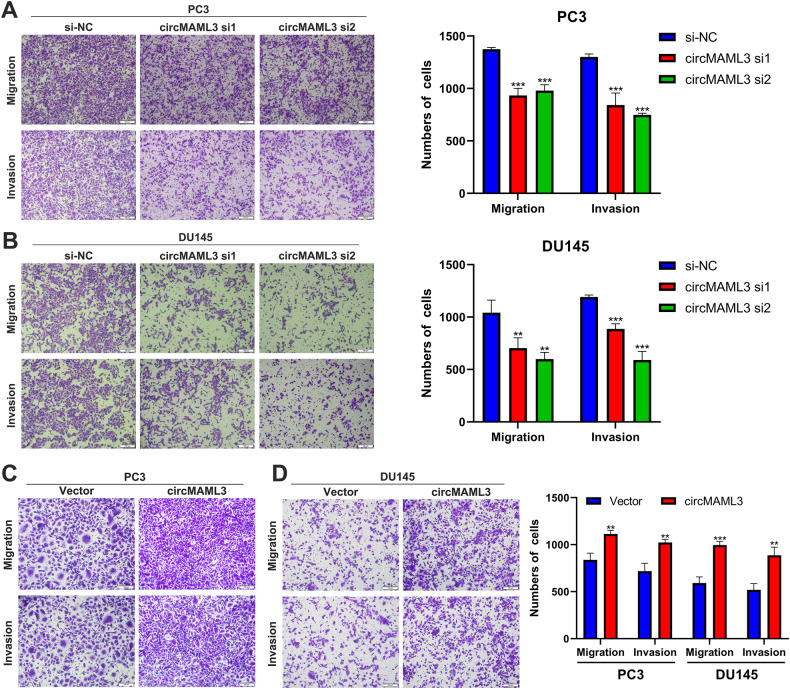
Transwell assays analysis of the effects of circMAML3 knockdown and overexpression on the migration and invasion of prostate cancer cells. **A**, **B** Knockdown of circMAML3 inhibits PC3 cell (**A**) and DU145 cell (**B**) migration and invasion. **C**, **D** Overexpression of circMAML3 promotes PC3 cell (**C**) and DU145 cell (**D**) migration and invasion. ** *P* < 0.01, *** *P* < 0.001.

We analyzed the expression of circMAML3 in cell lines and tissues by qRT-PCR and found that the expression level of circMAML3 in PCa cell lines was significantly higher than that in normal prostate epithelial cells (Fig. 2A), and circMAML3 expression in prostate cancer tissues was also significantly higher than that in normal tissues (Fig. 2B). To investigate circMAML3 function in prostate cancer, we transfected overexpressing circMAML3 lentivirus and siRNAs of knockdown circMAML3 into PC3 and DU145 cells. The results showed that circMAML3 was successfully overexpressed in the overexpression group compared with the vector group, while there was no significant change in MAML3 mRNA expression level (Fig. 2C, D); and the siRNA group (si1, si2 and si3) successfully suppressed circMAML3 expression compared to the si-NC group, whereas the MAML3 mRNA expression level was not significantly changed by the action of si1 and si2 (Fig. 2E, F). CCK-8 (Fig. 2G–J) and clone formation (Fig. 2K, L) assays showed that knockdown of circMAML3 resulted in decreased proliferative capacity of PC3 and DU145 cells, and conversely, overexpression of circMAML3 promoted proliferation of PC3 and DU145 cells. Furthermore, we assessed the impact of circMAML3 on the migratory and invasive capabilities of PCa cells through transwell assays. We found that knocking down circMAML3 significantly reduced the migratory and invasive abilities of PC3 and DU145 cells (Fig. 3A, B). On the other hand, overexpression of circMAML3 enhanced PC3 and DU145 cell migration and invasion (Fig. 3C, D).

### CircMAML3 functions as a sponge of miR‑665

**Fig. 4:**
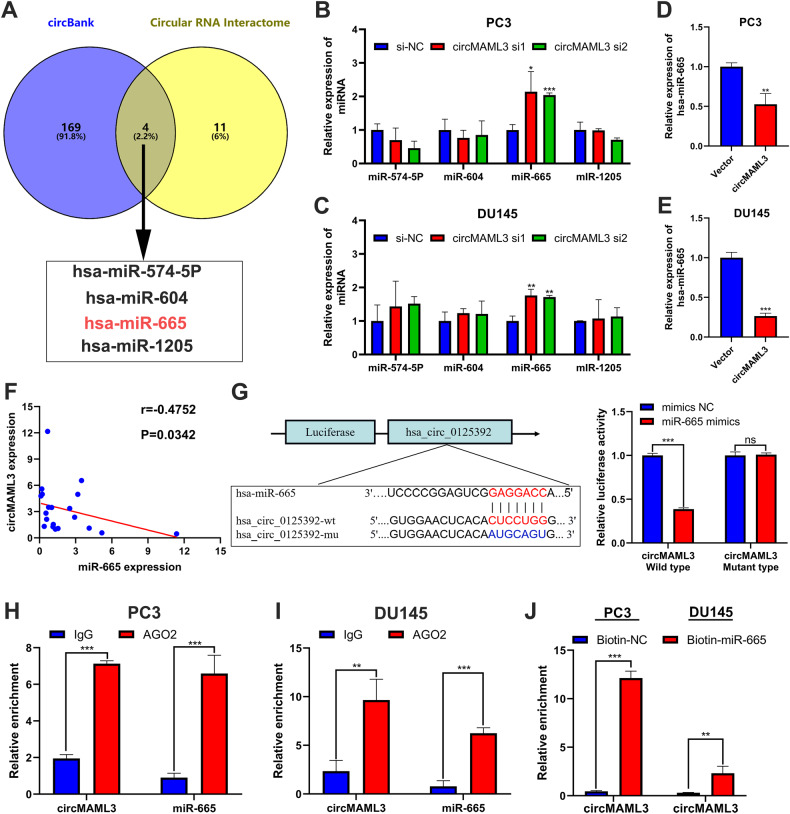
Predict and validate circMAML3 sponge miRNA. **A** circBank and circular RNA Interactome predict circMAML3 targeting downstream miRNAs. **B**, **C** qRT-PCR detection of the expression changes of miR-574-5p, miR-604, miR-665, and miR-1205 in PC3 (**B**) and DU145 (**C**) cells after knockdown circMAML3. **D**, **E** qRT-PCR detection of the expression changes of miR-665 in PC3 (**D**) and DU145 (**E**) cells after overexpression circMAML3. **F** qRT-PCR to detect the correlation between circMAML3 and miR-665 in 20 prostate cancer tissues. **G** Luciferase reporter assay was performed to determine the effect of miR-665 mimics on the luciferase activity of 293T cells transfected with circMAML3 wild type and circMAML3 mutant type. **H**, **I** RIP experiments were performed in PC3 (**H**) and DU145 (**I**) cells and confirmed by qRT-PCR assay that AGO2 significantly enriched circMAML3 and miR-665 compared to IgG. **J** miRNA pulldown experiments in PC3 and DU145 cells confirmed that biotin-miR-665 significantly pulled down circMAML3 compared to biotin-NC. * *P* < 0.05, ** *P* < 0.01, *** *P* < 0.001.

CircMAML3 is predominantly localized in the cytoplasm, according to the previous results. Therefore, we speculate that circMAML3 may function as a miRNA sponge in prostate cancer. To identify the miRNAs targeted by circMAML3, we performed predictions using circBank (http://www.circbank.cn/) [16] and circular RNA Interactome (https://circinteractome.irp.nia.nih.gov/) [17] databases. And by taking the intersection based on the prediction results, we identified four candidate miRNAs: miR-574-5p, miR-604, miR-665, and miR-1205 (Fig. 4A). After the knockdown of circMAML3 in PC3 and DU145 cells, only miR-665 expression was significantly upregulated among these four miRNAs (Fig. 4B, C). We further overexpressed circMAML3 in PC3 and DU145 and found decreased miR-665 expression (Fig. 4D, E). In addition, we found a significant negative correlation (r = −0.4752, *P* = 0.0342) between the expressions of circMAML3 and miR-665 in 20 PCa tissues (Fig. 4F). Next, we performed a dual-luciferase reporter assay to validate the interaction between circMAML3 and miR-665. The results demonstrated that miR-665 mimics significantly reduced the luciferase activity of the wild-type circMAML3 in 293T cells while showing no significant effect on the luciferase activity of the mutant-type circMAML3 (Fig. 4G). We also confirmed through the anti-AGO2 RIP assays that circMAML3 and miR-665 are significantly enriched in PC3 and DU145 cells compared to the IgG control antibody (Fig. 4H, I). Finally, we conducted miRNA pulldown assays using a biotin-labeled miR-665 probe to further investigate the interaction between miR-665 and circMAML3. The results revealed a significant enrichment of circMAML3 with biotin-miR-665 compared to the biotin-NC (Fig. 4J).

### CircMAML3 targets miR-665 to promote oncogenic effects in PCa cells

**Fig. 5:**
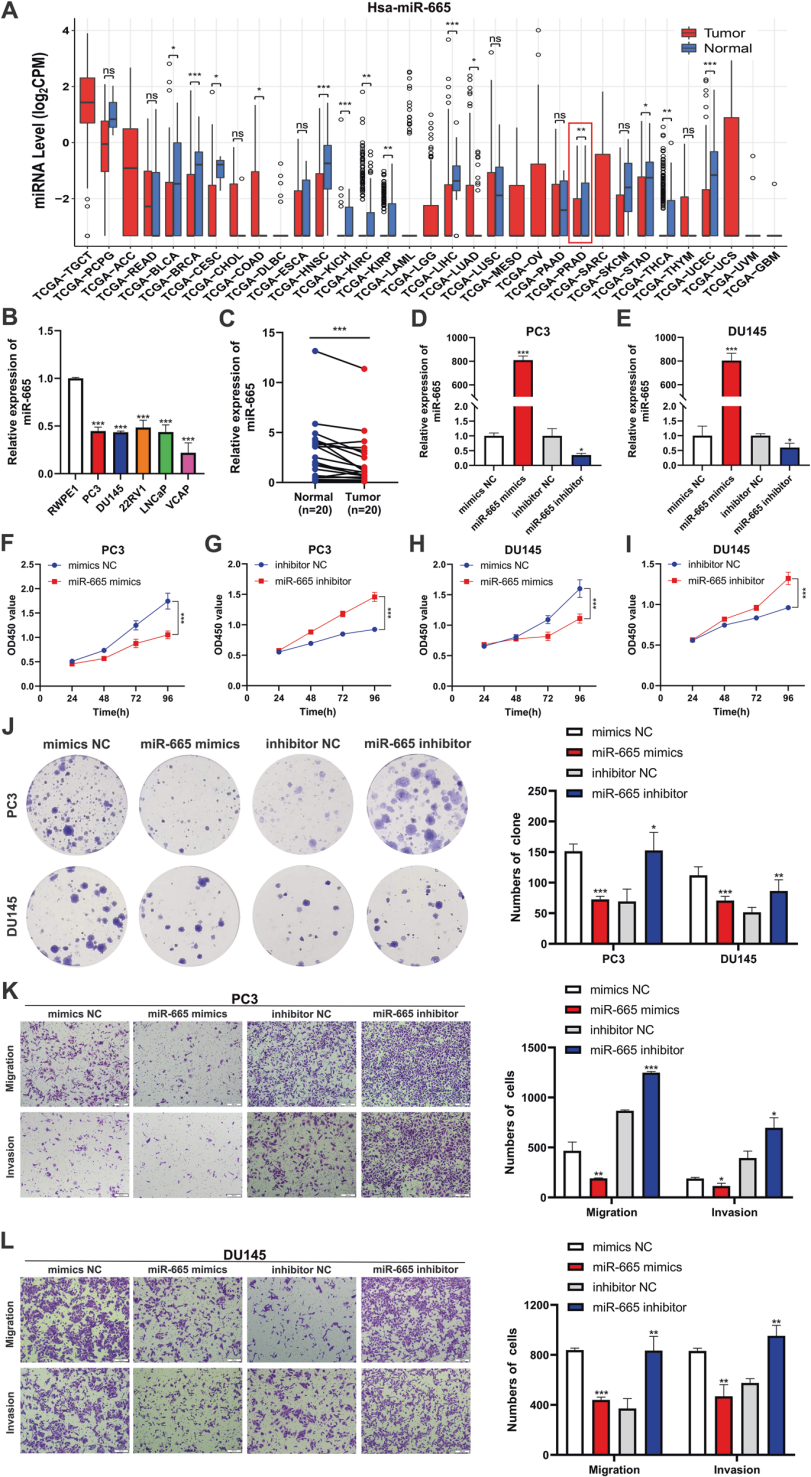
The expression of miR-665 in prostate cancer and its impact on the biological behavior of prostate cancer. **A** The online database CancerMIRNome was used to analyze the expression of miR-665 in pan-cancer. **B**, **C** qRT-PCR assay revealed that miR-665 was lowly expressed in prostate cancer cell lines (**B**) and prostate cancer tissues (**C**). **D**, **E** qRT-PCR was performed to detect miR-665 expression levels after transfection with miR-665 mimics or inhibitors in PC3 cells (**D**) and DU145 cells (**E**). **F**–**I** CCK-8 assay was conducted to assess the impact of transfecting miR-665 mimics and inhibitors on the proliferation of PC3 and DU145 cells. **J** Clone formation assays were performed to assess the impact of transfecting miR-665 mimics and inhibitors on the proliferation of PC3 and DU145 cells. **K**, **L** Transwell assays were performed to detect the effects of transfection with miR-665 mimics and inhibitors on the migration and invasion of PC3 (**K**) and DU145 (**L**) cells. * *P* < 0.05, ** *P* < 0.01, *** *P* < 0.001.

We found, through data analysis using CancerMIRNome (http://bioinfo.jialab-ucr.org/CancerMIRNome/) [18], that the expression level of miR-665 is significantly lower in most cancer tissues compared to normal tissues, including PCa (Fig. 5A). Furthermore, the expression level of miR-665 is significantly lower in PCa tissues and cell lines compared to their corresponding non-tumor tissues and normal prostate epithelial cells (Fig. 5B, C). Then, we constructed cell lines with overexpression and knockdown of miR-665 (Fig. 5D, E). The CCK-8 assay results indicated that miR-665 mimics significantly inhibited the proliferation of PC3 and DU145 cells (Fig. 5F, H), while the miR-665 inhibitor markedly increased the proliferative capacity of PC3 and DU145 cells (Fig. 5G, I). Similar results were obtained in the colony formation assay (Fig. 5J). In addition, the effects of miR-665 on the migration and invasion capabilities of prostate cancer cells were evaluated through the Transwell assay. Following the transfection of miR-665 mimics into PC3 cells, their migration and invasion abilities were significantly suppressed. Conversely, transfection of miR-665 inhibitor into PC3 cells led to a significant enhancement in migration and invasion capabilities (Fig. 5K). Similar results were observed in the Transwell assay conducted with DU145 cells (Fig. 5L).

**Fig. 6:**
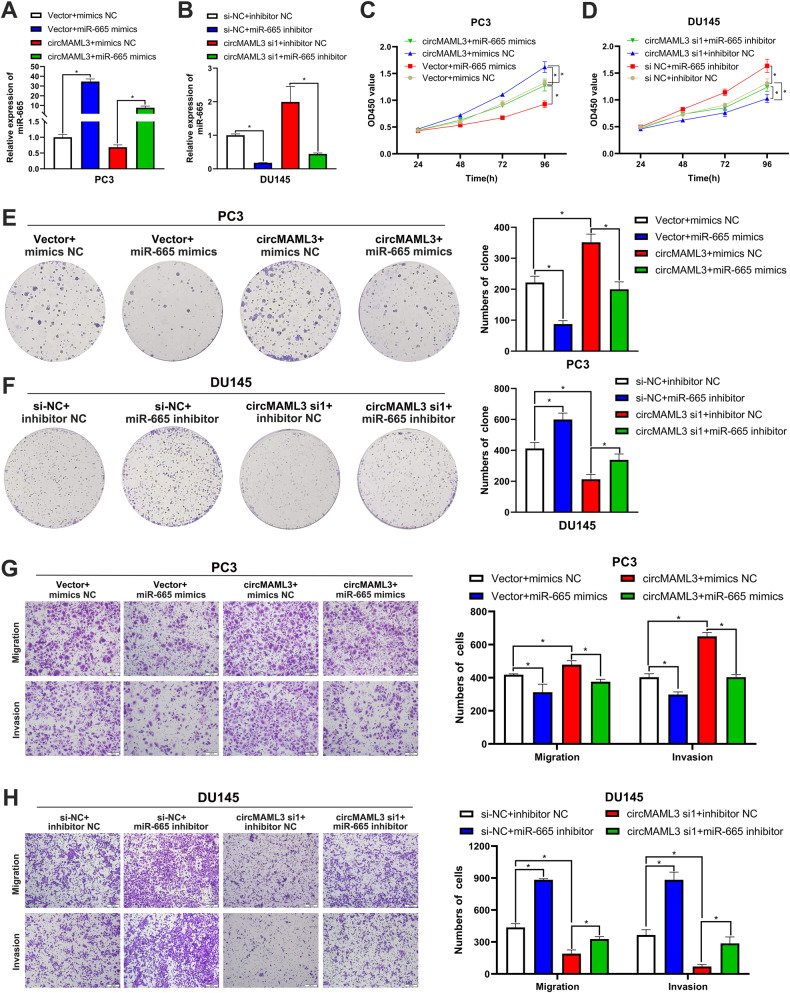
miR-665 can reverse the effects of circMAML3 on the biological behavior of prostate cancer. **A** qRT-PCR was performed to detect the expression changes of miR-665 in PC3 cells of overexpression circMAML3 and vector after transfected with miR-665 mimics and mimics NC. **B** qRT-PCR was conducted to assess the alterations of miR-665 expression in DU145 cells after co-transfection of si-NC/circMAML3 si1+inhibitor NC/miR-665 inhibitor. **C**, **E**, **G** CCK-8 assay (**C**), colony formation assay (**E**), and Transwell assay (**G**) were conducted to assess the impact for cell proliferation, migration, and invasion capabilities after transfecting with miR-665 mimics and mimics NC into PC3 cells of overexpression circMAML3 and Vector. **D**, **F**, **H** CCK-8 assay (**D**), colony formation assay (**F**), and Transwell assay (**H**) were conducted to assess the impact on cell proliferation, migration, and invasion capabilities after co-transfection of si-NC/circMAML3 si1+inhibitor NC/miR-665 inhibitor into DU145 cell. * *P* < 0.05.

To further investigate the function of miR-665 and circMAML3 in PCa, rescue assays were conducted. In PC3 cells with circMAML3 overexpression, the expression level of miR-665 was significantly increased after transfection with miR-665 mimics (Fig. 6A); On the other hand, in DU145 cells cotransfected with circMAML3 si1 and miR-665 inhibitor, the expression level of miR-665 was significantly downregulated (Fig. 6B). CCK-8 and clone formation assays demonstrated miR-665 mimics could reverse the promoting effect of circMAML3 overexpression on the proliferation ability of PC3 cells (Fig. 6C, E), and the suppressive effect of DU145 cell proliferation caused by circMAML3 knockdown could be reversed by miR-665 inhibitor (Fig. 6D, F). Similarly, the transwell assays also revealed that miR-665 mimics attenuated the promoting effect of circMAML3 overexpression on the migration and invasion abilities of PC3 cells (Fig. 6G), and miR-665 inhibitor significantly reversed the inhibitory effect of circMAML3 downregulation on the proliferative capacity of DU145 cells (Fig. 6H).

### MAPK8IP2 is directed by miR-665 targeting and indirectly regulated by circMAML3

**Fig. 7:**
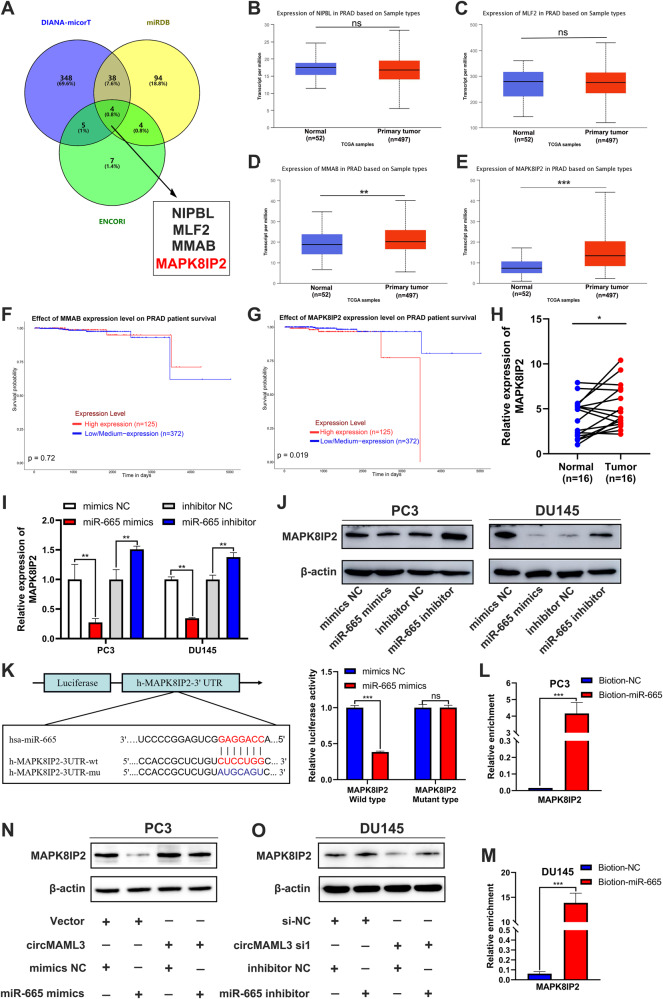
Prediction and validation of miR-665-targeted mRNA. **A** DIANA-microT, miRDB and ENCORI databases predict miR-665 targeting downstream mRNAs. **B**–**E** UALCAN database analysis of NIPBL (**B**), MLF2 (**C**), MMAB (**D**) and MAPK8IP2 (**E**) expression in prostate cancer tissues versus normal tissues. **F**, **G** Evaluate the survival of prostate cancer patients in MMAB (**F**) and MAPK8IP2 (**G**) high-expression tissues compared to the low-expression group using the UALCAN database. **H** qRT-PCR detection of MAPK8IP2 expression in 16 pairs of matched prostate cancer tissues and normal tissues. **I**, **J** qRT-PCR (**I**) and western blot (**J**) analysis of the impact of transfection with miR-665 mimics and inhibitor on MAPK8IP2 mRNA expression levels in PC3 and DU145 cells. **K** Luciferase reporter assay was performed to determine the effect of miR-665 mimics on the luciferase activity of 293T cells transfected with MAPK8IP2 wild type and circMAML3 mutant type. **L**, **M** miRNA pulldown experiments in PC3 (**L**) and DU145 (**M**) cells confirmed that biotin-miR-665 significantly pulled down MAPK8IP2 compared to biotin-NC. **N** Western blot analysis indicates that miR-665 mimics can rescue the elevated levels of MAPK8IP2 protein caused by circMAML3 overexpression in PC3 cells. **O** Western blot analysis of the impact on MAPK8IP2 protein levels after co-transfection of si-NC/circMAML3 si1 and inhibitor NC/miR-665 inhibitor in DU145 cells. ns: not significant, * *P* < 0.05, ** *P* < 0.01, *** *P* < 0.001.

For miR-665 target regulation of genes in prostate cancer is not clear. Therefore, the online databases DIANA-microT [19], miRDB [20], and ENCORI [21] were utilized to predict the mRNA targets regulated by miR-665. By taking the intersection of the results, four potential mRNA targets were identified, namely NIPBL, MLF2, MMAB, and MAPK8IP2 (Fig. 7A). We further analyzed using the UALCAN [22] database and found that only high expression of MAPK8IP2 is associated with poor survival in PCa patients (Fig. 7B–G). At the same time, qRT-PCR analysis also revealed that the expression level of MAPK8IP2 is significantly higher in PCa tissues compared to their corresponding non-tumor tissues (Fig. 7H). Therefore, we consider that MAPK8IP2 may serve as a downstream target gene regulated by miR-665 to influence prostate cancer biological behavior.

Further, qRT-PCR and western blot analysis revealed that transfection of miR-665 mimic significantly downregulates the expression of MAPK8IP2 in PC3 and DU145 cells, and transfection of miR-665 inhibitor leads to a significant increase in MAPK8IP2 expression (Fig. 7I, J). The dual-luciferase reporter assay showed that miR-665 mimics significantly reduce the wild-type MAPK8IP2 luciferase activity in 293T cells while having no significant effect on the mutant-type MAPK8IP2 luciferase activity (Fig. 7K). The miRNA pulldown assay also demonstrated a significant enrichment of MAPK8IP2 by biotin-miR-665 compared to biotin-NC in PC3 and DU145 cells (Fig. 7L, M). In addition, miR-665 mimics or inhibitors can reverse the alterations in MAPK8IP2 caused by overexpression or knockdown of circMAML3 (Fig. 7N, O).

**Fig. 8:**
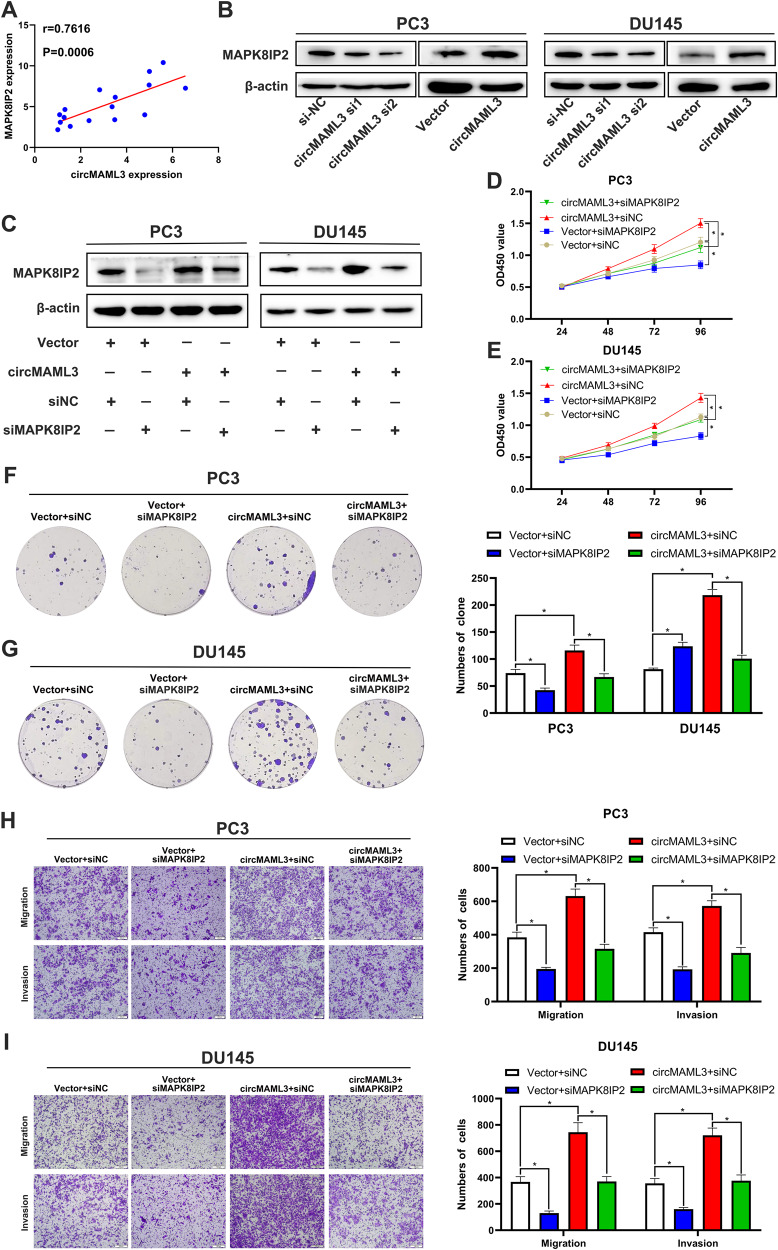
circMAML3 is associated with MAPK8IP2 expression and downregulation of MAPK8IP2 reverses the role of circMAML3 in promoting prostate cancer progression. **A** Pearson correlation analysis indicates a significant positive correlation between circMAML3 and MAPK8IP2. **B** Western Blot analysis to assess the effect of knockdown (or overexpression) of circMAML3 on MAPK8IP2 protein expression in PC3 and DU145 cells. **C** Western blot analysis to assess MAPK8IP2 protein expression after transfected with siMAPK8IP2 in PC3 and du145 cells of overexpression circMAML3. **D**, **E** CCK-8 assays demonstrated that knocking down MAPK8IP2 could restore the increased proliferative capacity of PC3 cells (**D**) and DU145 cells (**E**) caused by overexpression of circMAML3. **F**, **G** Clone formation assays showed that knockdown of MAPK8IP2 restored the enhanced proliferative capacity of PC3 (**F**) and DU145 (**G**) cells caused by overexpressing circMAML3. **H**, **I** Transwell assay analysis revealed that knocking down MAPK8IP2 could restore the enhanced migration and invasion capacity of PC3 cells (**H**) and DU145 cells (**I**) induced by overexpression of circMAML3. * *P* < 0.05.

The qRT-PCR results indicate a significant positive correlation between the expression level of circMAML3 and MAPK8IP2 in PCa tissues (r = 0.7616, *P* = 0.0006) (Fig. 8A). In PC3 and DU145 cells, overexpression of circMAML3 leads to increase MAPK8IP2 expression, whereas knockdown of circMAML3 caused downregulation of MAPK8IP2 expression (Fig. 8B). Further rescue assays indicated that siMAPK8IP2 could reverse the downregulation of MAPK8IP2 caused by overexpression of circMAML3 (Fig. 8C). The function rescue assays of CCK-8, clone formation and transwell assays showed that siMAPK8IP2 reversed the increased proliferation, migration and invasion ability of PC3 and DU145 cells caused by overexpression of circMAML3 (Fig. 8D–I).

### CircMAML3 facilitates the progression of PCa in vivo

**Fig. 9:**
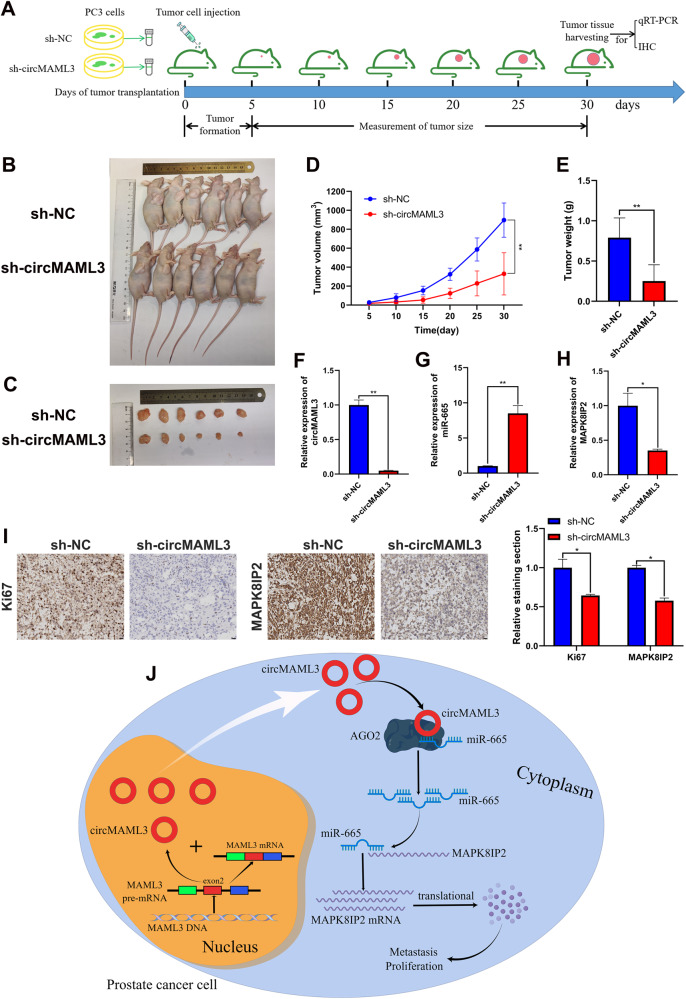
The effect of knockdown circMAML3 on prostate cancer cell growth in vivo. **A** Subcutaneous tumor formation process in nude mice. **B** Subcutaneous burden tumor model in nude mice. **C** Comparison of tumor size in vitro between sh-NC group and sh-circMAML3 group. **D** sh-NC group and sh-circMAML3 group tumorigenic growth change curves in vivo. **E** Comparison of tumor weight between sh-NC group and sh-circMAML3 group. **F**–**H** qRT-PCR Detection of circMAML3 (**F**), miR-665 (**G**), and MAPK8IP2 (**H**) expression in tumor tissues between the sh-NC group and sh-circMAML3 group. **I** Immunohistochemical analysis of Ki67 and MAPK8IP2 expression in the tumors of the sh-NC group versus the sh-circMAML3 group (scale bar = 20 µm). **J** Schematic diagram of the mechanism by which circMAML3 promotes prostate cancer progression via regulation of the miR-665/MAPK8IP2 axis (this figure was created using the figdraw platform). * *P* < 0.05, ** *P* < 0.01.

To assess the biological effects of circMAML3 in vivo, we subcutaneously injected PC3 cells stably transfected with sh-NC and sh-circMAML3 into BABL/c nude mice. Subcutaneous tumorigenesis experiments in nude mice were performed, as shown in Fig. 9A, and the results indicated that the knockdown of circMAML3 inhibited the growth of PCa cells in vivo (Fig. 9B–E). qRT-PCR assay revealed that circMAML3 and MAPK8IP2 of the sh-circMAML3 group were lower compared to the sh-NC group, in contrast, miR-665 expression levels were higher in the sh-circMAML3 group than in the sh-NC group (Fig. 9F–H). The immunohistochemistry results indicated that the staining of Ki67 and MAPK8IP2 was significantly weaker in the sh-circMAML3 group compared to the sh-NC group (Fig. 9I).

## Discussion

After more than a decade of in-depth exploration of circRNA, an increasing number of studies have demonstrated that circRNA holds significant potential to serve as biomarkers for tumor diagnosis and prognosis, as well as therapeutic targets [23]. Despite numerous studies reporting and exploring the biological roles and mechanisms of circRNAs in PCa [24]. there are still a large number of aberrantly expressed circRNAs in PCa that have not been explored.

In this study, we discovered that circMAML3 is highly expressed in PCa. Through in vitro cellular experiments, we confirmed that circMAML3 is a stably expressed circular transcript in PCa cells, predominantly located in the cytoplasm. The occurrence and progression of tumors are closely associated with aberrant proliferation, metastasis, and invasion [25]. Many studies have reported that circRNAs promote or inhibit tumor proliferation, migration and invasion. Shi et al. [26] reported that circANAPC7 suppresses pancreatic proliferation. CircSPARC was reported to promote proliferation and metastasis in colorectal cancer [27]. CircBCAR3 facilitates tumorigenesis and metastasis in esophageal cancer [28]. Circ_0001686 has been identified to enhance prostate cancer proliferation, migration and invasion [29]. Through a series of in vitro and in vivo experiments, we have confirmed that circMAML3 plays a role in promoting prostate cancer cell proliferation, migration, and invasion.

Previous studies have shown that circRNAs regulate cancer progression through different pathways, such as circRNAs interacting with RNA polymerase II (Pol II) to regulate transcription and splicing of parental genes; circRNAs acting as protein scaffolds and recruitment proteins; circRNA “sponges” miRNAs, or protein; and circRNAs being driven by m6A methylation or IRES to translate proteins [30]. CircRNAs localized in the cytoplasm mainly block miRNAs from interacting with their target genes by acting as miRNA molecular sponges [31]. As a classic study of miRNA molecular sponges, ciRS-7 was the first to functionally analyze naturally expressed circRNAs. ciRS-7 has more than 60 miR-7 binding sites, and up-regulates the expression of miR-7 target genes by binding to miR-7 [32]. Yu et al. [11] found that hsa_circ_0003258 was mainly localized in the cytoplasm of prostate cancer cells and could promote prostate cancer migration and invasion by “sponging” miR-653-5P to upregulate the expression of ARHGAP5. Therefore, we hypothesize that circMAML3 primarily exerts its function in the cytoplasm by acting as a “sponge” of miRNAs. The correlation analysis of circMAML3 and miR-665 expression in PCa tissues, RIP assay, miRNA pulldown assay and dual-luciferase reporter assay revealed that circMAML3 could directly bind to miR-665.

MiR-665, as a member of the miRNA family, has been demonstrated to be involved in the regulation of various cancers. On the one hand, miR-665 has been reported to play a pro-carcinogenic role in tumors. miR-665 targets TRIM8 to regulate the Wnt5a/β-Catenin and Caspase-3 signaling pathways in lung squamous carcinoma to promote cell proliferation and inhibit apoptosis [33]; miR-665 is associated with poor prognosis and promotes metastasis by targeting NR4A3 in breast cancer [34]. On the other hand, miR-665 was found to play an anti-cancer role in tumors. MiR-665 exerts its tumor-suppressive role in gastric cancer by targeting different mRNAs [35–37]. miR-665 inhibits epithelial-mesenchymal transition and suppresses progression by blocking the SMAD3/SNAIL axis in bladder cancer [38]. We confirmed that miR-665 plays a tumor-suppressive role in PCa by cell function assays. The results of rescue experiments indicated that miR-665 mimics or inhibitors could restore the effects of overexpression or knockdown of circMAML3 on PCa cells. Overall, our results suggest that circMAML3 can sponge miR-665 involved in PCa proliferation, migration and invasion. In addition, we further identified MAPK8IP2 as a downstream target gene of miR-665 by bioinformatics analysis, qRT-PCR, dual-luciferase reporter assay and miRNA pulldown assay.

MAPK8IP2 is located on chromosome 22 (22q13) and has a structure similar to that of JIP1, including the SH3, the PTB, and the JNK-binding structural domain, which are involved in the regulation of the JNK and P38 MAPK signaling pathways [39–41]. Only a few studies have reported the role of MAPK8IP2 in cancer. Recent studies have found that MAPK8IP2 may be associated with the prognosis of glioblastoma and pancreatic cancer [42, 43]. In addition, MAPK8IP2 was identified to be associated with cervical cancer progression [44]. MAPK8IP2 expression was associated with AR signaling activity in PCa [45]. In previous studies, we confirmed the upregulation of MAPK8IP2 expression in PCa and its role in promoting the progression of PCa [46]. Through a series of experiments, we determined that circMAML3 promotes PCa progression by sponge miR-665 upregulation of MAPK8IP2 expression.

## Conclusions

Collectively, this study was the first to identify the biological roles of circMAML3, miR-665 and MAPK8IP2 in prostate cancer, which clearly demonstrated that circMAML3 and MAPK8IP2 play an oncogenic role in prostate cancer, and miR-665 inhibits the progression of PCa, and it was confirmed that circMAML3 promotes the progression of PCa by targeting miR-665 to upregulate the expression of MAPK8IP2 (Fig. 9J).

## Materials and methods

### Tissue sample acquisition

Tumor tissue and adjacent normal tissue were obtained from 20 PCa patients undergoing radical prostatectomy in the First Affiliated Hospital of Nanchang University. Inclusion criteria: (1) All organizations are diagnosed independently by two pathologists; (2) Patients did not receive relevant anti-tumor treatment before surgery. Exclusion criteria: (1) Patients with other primary tumor diseases; (2) Pathological diagnosis is non-prostate adenocarcinoma. The ethics committee of the First Affiliated Hospital of Nanchang University approved the tissue collection and related experiments of this study (approval no. 2017 [075]). All patients signed informed consent.

### Cell culture

The human PCa cell lines (LNCaP, VCaP, 22RV1, DU145, and PC3) and the human normal prostate epithelial cell lines (HPrEC and RWPE-1) were purchased from ATCC. All the cells were incubated in a 37 °C incubator containing 5% CO_2_. Cells were cultured in different media, PC3 cells using F12K medium (Gibco, USA), HPrEC cells using prostate epithelial cell basal medium (ATCC, USA), RWPE-1 and VCaP cells using DMEM (Gibco, USA), and DU145, 22RV1, and LNCaP cells using RPMI-1640 medium (Gibco, USA). All media contain 10% FBS (Gibco, Australia).

### RNA and genomic DNA (gDNA) extraction

Total RNA of tissues and cells extraction was performed using TRIzol reagent (CWBIO, China), and the First-Strand cDNA Synthesis kit (Transgen, AT341-01, China) was used for reverse transcribed into cDNA. gDNA was extracted by *EasyPure*^®^ Genomic DNA Kit (Transgen, EE101-01, China) according to the manufacturer’s protocols.

### Quantitative real‑time PCR (qRT-PCR)

The TransStart Green qPCR SuperMix kit (Transgen, AQ101-01, China) was used for qRT-PCR. The Bulge-Loop™ miRNA qRT-PCR Primer Sets were obtained from RIBOBIO (Guangzhou, China) for miRNA qRT-PCR. β-actin as a circRNA/mRNA reference gene and U6 as a miRNA reference gene, calculate the relative expression using the method of 2^−^^∆∆CT^. The primer sequences of circRNA/mRNA are listed in Supplementary Table 1.

### RNase R and actinomycin D treatment

Total RNA (4 µg) from PC3 and DU145 cells was treated with or without 2U/µg RNase R (GENESEED, China) at 37 °C for 15 min. PC3 and DU145 cells were treated with 2 µg/ml actinomycin D (Sigma, USA) for 0, 3, 6, 9, 12 and 24 h to extract RNA for qRT-PCR analysis.

### Nuclear‑cytoplasmic fractionation

We isolated cytoplasmic and nuclear RNA using NE-PER™ Nuclear and Cytoplasmic Extraction Reagent (Thermo Fisher Scientific, USA). β-actin as a cytoplasmic reference gene and U6 as a nuclear reference gene. Further analysis was performed by qRT-PCR.

### Fluorescence in situ hybridization (FISH)

FISH experiment was performed to visualize the subcellular localization of circMAML3 in PCa cells. Cy3-SA-Biotin-labeled circMAML3 (GenePharma, China) probe was synthesized to capture circMAML3. RNA FISH SA-Biotin Amplification System Kit (GenePharma, China) was obtained for FISH assay, following the manufacturer’s instructions. The images were observed by confocal laser microscopy (Leica, Germany). The probe sequence is as follows: Biotin-CTGACCGTGGGATGGACACAGTCTCTTGTAG.

### Cell transfection

miR-665 mimics and inhibitors were synthesized by RiboBio (Guangzhou, China). The sequence of siMAPK8IP2 was described in a previous study [46]. The siRNA of circMAML3, overexpression or knockdown lentivirus of circMAML3 designed and synthesized by Hanbio (Shanghai, China). The siRNA, miR-665 mimics and miR-665 inhibitor were transfected into PC3 and DU145 cells using lip2000 according to the manufacturer’s guidelines. Lentivirus-infected PC3 and DU145 cells were screened by puromycin (6 μg/ml) for 48 h to obtain stably expressed cell lines and maintained in culture at puromycin (1 µg/ml). The sequences of circMAML3 siRNA are listed in Supplementary Table 2.

### Cell proliferation assays

CCK-8 and clone formation assays were used to assess the effect of circMAML3 on the proliferation of prostate cancer cells. ccK-8 assays were performed as previously reported [46]. For clone formation, the transfected cells were seeded in a six-well plate, with 800 cells per well, and cultured in a complete growth medium for a duration of 10 days. Subsequently, they were fixed using a 4% paraformaldehyde solution for 30 min and stained with 1% crystal violet for another 30 min. Following staining, the cells were thoroughly rinsed with tap water and quantified utilizing ImageJ software for counting analysis.

### Transwell migration and invasion assays

Transwell migration and invasion assays were as described in a previous study [46].

### Luciferase reporter assay

The circMAML3 and MAPK8IP2 wild-type and mutant sequences associated with the miR-665 binding site were cloned into a dual-luciferase reporter vector of pSI-Check2 named by Hanbio (Shanghai, China). The dual luciferin reporter vector and miR-665 mimics were cotransfected into 293T cells, and after 48 h, measure the activity of firefly luciferase and Renilla luciferase using a dual-luciferase reporter assay kit (Hanbio, China) according to the manufacturer’s instructions.

### RNA immunoprecipitation (RIP) assay

We performed RIP assays using the BersinBio^TM^ RIP Kit (BerSinBio, # Bes5101) with AGO2 antibody (Proteintech, #67934-1-Ig) according to the manufacturer’s instructions.

### Biotinylated miRNA pulldown assay

Biotin-labeled miR-665 and miR-NC probes (Genepharma, 100 nM) were transfected into PC3 and DU145 cells. Perform the experiment according to the miRNA pulldown kit (BersinBio, China) guidelines. Briefly, cells transfected for 48 h were collected and lysed. Subsequently, the lysate was mixed with streptavidin magnetic beads and incubated overnight at 4 °C with rotation. Finally, the eluted RNA was purified and utilized for qRT-PCR analysis to ascertain the relative enrichment levels of circMAML3 and MAPK8IP2. The probe sequences were as follows: miR-665 (5′-3′): ACCAGGAGGCUGAGGCCCCU-Biotin; miR-NC (5′-3′): UUGUACUACAAAAG UACUG- Biotin.

### Western blot assay

Extract total protein using RIPA lysis buffer (APExBIO, USA) and quantify the protein using a BCA kit (CWBIO, China). Separate proteins on a 10% SDS-PAGE gel followed by transfer to a PVDF membrane. Block the membrane in 5% skim milk for 1 h at room temperature and proceed with primary antibody incubation overnight at 4 °C. Finally, after incubating with the secondary antibody at room temperature for 1 h, visualization was achieved using enhanced chemiluminescence. The primary antibodies include: β-actin (1:1000, #TA811000, ORIGEN, USA) and MAPK8IP2 (1:500, #PA5-101157, Termo Fisher Scientifc, USA).

### Animal models

The animal experiments were approved by the Experimental Animal Welfare Ethics Committee of the First Affiliated Hospital of Nanchang University. BALB/C male nude mice (4 weeks old) were purchased from Ziyuan Laboratory Animal Technology Co. (Hangzhou, China). PC3 (5 × 10^6^/ per mouse) cells transfected with LV-sh-NC or LV-sh-circMAML3 were resuspended in a 200 μl mixture (equal volumes of F12K medium and Matrigel) and injected subcutaneously into the right flank of mice. The growth status of mice was observed and tumor volume was measured every 5 days. The tumor volume was calculated using the formula: volume = (π × length × width^2^/6).

### Immunohistochemistry (IHC)

The obtained subcutaneous tumor tissues of nude mice were paraffin-embedded and sectioned. The sections were dewaxed, hydrated, antigen retrieved and blocked. Subsequently, they were incubated overnight with the primary antibodies Ki67 (Servicebio, China) and MAPK8IP2 at 4 °C, followed by incubation with secondary antibody for 1 h at room temperature. Lastly, diaminobenzidine and hematoxylin staining were performed. The images were taken with a Zeiss microscope.

### Statistical analysis

GraphPad Prism 9.0.0 was used for graphing and statistical analysis in this study. For two groups of data that are normally distributed and have equal variances, we employed either a paired or unpaired Student’s *t*-test. If the variances were found to be unequal, a corrected *t*-test (Welch’s test) was utilized. In cases involving comparisons between multiple groups, we conducted either a one-way or two-way analysis of variance (ANOVA). For non-normally distributed data, non-parametric tests were employed for analysis. Correlation analysis for data that followed a normal distribution was performed using Pearson’s correlation coefficient, whereas, for data that did not follow a normal distribution, Spearman’s correlation coefficient was utilized. A significance level of *P* < 0.05 was considered statistically significant.

### Supplementary information

supplementary table1

supplementary table2

## Data Availability

All experiment data are available from the corresponding author upon reasonable request.
