# Supplementary material for: A novel circular RNA, circMAML3, promotes tumor progression of prostate cancer by regulating miR-665/MAPK8IP2 axis
Source: Cell Death Discov. 2023 Dec 14;9:455. doi: 10.1038/s41420-023-01750-1 (PMC10721837; doi:10.1038/s41420-023-01750-1)
Supplement: Supplementary file 1 — supplementary table1 [file 41420_2023_1750_MOESM1_ESM.docx]

Table S1 The primer sequences for qRT-PCR

| Gene | Forward (5′-3′) | Reverse (5′-3′) |
| --- | --- | --- |
| circMAML3  MAML3 | CTTACGCTGCACTTCCATCC  CCCTTGCAGAACAGTGGAAC | AGTGGTGATCGAGCTCCTTC  GCAACCGTTGACAGGAAGTT |
| MAPK8IP2 | CGCTGCAGCCATTTCTTCC | ACTCCTGGGAGACAAAGACG |
| β-actin | TCTCCCAAGTCCACACAGG | GGCACGAAGGCTCATCA |
| U6 | AAAGCAAATCATCGGACGACC | GGGGTCGTTGATGGCAACA |
