# Supplementary material for: A novel circular RNA, circMAML3, promotes tumor progression of prostate cancer by regulating miR-665/MAPK8IP2 axis
Source: Cell Death Discov. 2023 Dec 14;9:455. doi: 10.1038/s41420-023-01750-1 (PMC10721837; doi:10.1038/s41420-023-01750-1)
Supplement: Supplementary file 2 — supplementary table2 [file 41420_2023_1750_MOESM2_ESM.docx]

|  | siRNA sequence |
| --- | --- |
| si-1 |  |
| sense (5′-3′) | CCCACGGUCAGCUACAAGATT |
| antisense (5′-3′) | UCUUGUAGCUGACCGUGGGTT |
| si-2 |  |
| sense (5′-3′) | CGGUCAGCUACAAGAGACUTT |
| antisense (5′-3′) | AGUCUCUUGUAGCUGACCGTT |
| si-3 |  |
| sense (5′-3′) | CAUCCCACGGUCAGCUACATT |
| antisense (5′-3′) | UGUAGCUGACCGUGGGAUGTT |
| si-NC |  |
| sense (5′-3′) | UUCUCCGAACGUGUCACGUTT |
| antisense (5′-3′) | ACGUGACACGUUCGGAGAATT |

Table S2 The sequences of circMAML3 siRNA
